# Preimplantation Factor (PIF): a peptide with various functions

**DOI:** 10.5935/1518-0557.20190082

**Published:** 2020

**Authors:** Fateme Zare, Seyed Mohammad Seifati, Mahdi Dehghan-Manshadi, Farzaneh Fesahat

**Affiliations:** 1 Reproductive Immunology Research Center, Shahid Sadoughi University of Medical Sciences, Yazd, Iran; 2 Department of Immunology, School of Public Health, Tehran University of Medical Sciences, Tehran, Iran; 3 Department of Immunology, School of Medicine, Mashhad University of Medical Sciences, Mashhad, Iran

**Keywords:** preimplantation factor, peptide, pregnancy, immune system

## Abstract

Preimplantation Factor (PIF) is a novel fifteen amino acid linear peptide (MVRIKPGSANKPSDD), which has different biological functions in mammalian species e.g. its role in neuron restoration, pregnancy and related disorders, and also in autoimmune diseases. Since all clinical studies have shown that PIF has both local and systemic effects, it can be considered as an integrated therapy for the treatment of inflammation conditions, along with the prevention of advanced disease. The synthetic PIF (sPIF) analog is a good representative of native PIF action, and it regulates peripheral immune cells to achieve endurance without immune suppression - an effective agent in nonpregnant autoimmune models. This study provides information, from evidence-based studies so far about PIF’s different functional aspects.

## INTRODUCTION

PIF was first introduced by Dr. Eytan Barnea, as a native peptide secreted only by permanent mammalian embryos, as early as the 2-cell stage to the fetus from the beginning to the end stage of viable pregnancy (Roussev *et al*., 1995). Later, the Barnea Research Group studied the potential therapeutic role of PIF against diseases and immune disorders associated with pregnancy, such as endometriosis, recurrent pregnancy loss (RPL), preeclampsia (Barnea *et al*. 2016a; Goodale *et al*., 2017; Sbracia *et al*., 2017). Consequently, PIF as a novel fifteen amino acid linear peptide (MVRIKPGSANKPSDD) has different biological functions in mammalian species, namely its role in neuron restoration, pregnancy and related disorders and also in autoimmune diseases (Barnea *et al*., 2015; Goodale *et al*., 2017). PIF showed multi-functional effects in decidua cultures, such as modulating local immunity and systemic maternal immunity without suppression, embryo adhesion enhancement, apoptosis control and trophoblast invasion induction (Duzyj *et al*., 2010; Paidas *et al*., 2010; Weiss *et al*., 2012).

Given the new knowledge about the role of fetal secretions, including PIF in embryo acceptance of the females' immune system throughout viable pregnancy, motherhood like the past is not considered as the only determinant of pregnancy success. In the current review, we attempt to describe the different functional aspects of PIF according to the literature.

## FUNCTIONS

### Neuron Restoration

PIF plays a major role in the embryo’s neural system development and neuroprotection by providing the embryo with proteins engaged in oxidative stress, protein misfolding, and neural development, attacking to protein-di-isomerase/thioredoxin (PDI/TRX) and heat shock proteins (HSPs) (Barnea *et al*., 2014). Moreover, PIF can target tubulins as well as the neurons’ backbone. It can also upregulate decidual proteins involved in neural function, including agrin as a part of the neuromuscular junction; Calpain1 as a cytoskeleton member; NADH dehydrogenase (ubiquinone); iron-sulfur protein 3 (NDUFS3), a modulator of oxidative stress; and protein-tyrosine phosphatase receptor-type F polypeptide-interacting binding protein 1 (PPF1BP1) as an axon guidance agent (Paidas *et al*., 2010).

PIF prevents severe brain damage caused by hypoxia and inflammation in the newborn animal model (Weiss *et al*., 2012). However, PIF facilitates neural repair via local and systemic effects, it suppresses severe paralysis through oxidative stress reduction and protein misfolding in chronic neuroinflammation models. Additionally, the subcutaneous implementation of PIF resulted in declined brain inflammation and increased neural repair in a clinical model of multiple sclerosis (MS) (Weiss *et al*., 2012). Interestingly, evidence has indicated that PIF targets microglia as the major immune element within the central nervous system. Following the effects of PIF on microglia culture, the reduction in a let-7 microRNA- dependent pathway involving Protein C/A (PKC/PKA) kinases demonstrated its neuro-protective influence on brain injury (Mueller *et al*., 2014; Mueller *et al*., 2015). A synthetic preimplantation factor (sPIF), analogous to PIF obtained from the mammalian embryos, can provide neuro-protection in rodent models of experimental autoimmune encephalomyelitis as well as perinatal brain injury (Weiss *et al*., 2012; Mueller *et al*., 2015).

### Role in pregnancy

PIF is a specific peptide secreted by vital embryos starting at a two-cell stage in mice, four-cell stage in humans, and six-cell stage in bovines. It presents its key role in early fetal as well as maternal signaling (Stamatkin *et al*., 2011a; Barnea *et al*., 2012a). PIF is acquirable in maternal serum of two days from mouse pregnancy as well as on 2-day post-embryo transfer in human *in vitro* fertilization (IVF) cycles, (Coulam *et al*., 1995; Roussev *et al.*, 1995).

PIF is detectable during the first trimester of pregnancies that improve to the second trimester (Barnea *et al*., 1994). Due to the pro-apoptotic effects of PIF, it can create a beneficial pro-inflammatory environment in human decidual cells (Moindjie *et al*., 2016). However, the absence of PIF declares no pregnancy in bovines, positive detection of PIF at day 20 after artificial insemination auspicates normal calf delivery (Ramu *et al*., 2013).

PIF is expressed by both the mammalian embryo and its placenta, which shows in maternal circulation with assured autotrophic effect on the embryo until term (Barnea, 2007; Barnea *et al*., 2012a). Unlike non-viable pregnancy, it is also shown in the maternal circulation throughout a viable pregnancy (Barnea, 2007; Stamatkin *et al*., 2011a; Ramu *et al*., 2013). There are four main supplementary effects of this peptide that are necessary for pregnancy initiation and maintenance, including:

### Embryo development and maintenance as a rescue factor

The development of in-vitro embryos depends on the autocrine factors they secrete (O’Neill, 1997; Gopichandran & Leese, 2006). Moreover, reactive oxygen species (ROS) are one of the main harmful agents affecting embryos growth that have to be reduced in-vitro (Takahashi, 2012; Latham, 2016). There are persistent concerns about multiple embryos culture for an extended period during IVF that can cause damaging epigenetic effects as well as premature delivery (Nasr-Esfahani *et al*., 1992). Viable embryos secrete PIF as an internal compound that increases their development and protection against adverse in-vitro conditions (Barnea *et al*., 1994; Roussev *et al*., 1995; Duzyj *et al*., 2010). A study conducted by Goodale *et al*. (2017) demonstrated that PIF postponed embryo development resulting from RPL attenuating ROS and concluded that the protein disulfide isomerase/thioredoxin (PDI/TRX) was considered as an initial PIF target as well as an important ROS scavenger in the embryo.

Some reports showed that the exogenous administration of sPIF has improved the rate of embryo viability and blastocyst developments in animal models in an autocrine and paracrine manner when compared to the controls (Barnea *et al*., 2014), while anti-PIF monoclonal antibody had inhibitory effects (Stamatkin *et al*., 2011a;b).

### Endometrial receptivity induction

In order to achieve a successful pregnancy, it seems to be necessary the simultaneous adoption of embryonic development and endometrial status during a receptive period known as the implantation window (Miravet-Valenciano *et al*., 2015).

PIF raises the implantation window and endometrial embryo receptibility, creating a pro-inflammatory situation that promotes embryo adhesion, and regulates apoptosis before the implantation, the responsibility of deciduas in the first trimester (Paidas *et al*., 2010). In this regard, it has been demonstrated the improved embryo receptivity induced by sPIF on decidualized human endometrial stromal cells (HESCs, an implantation model), as well as in deciduas in the first trimester (Barnea *et al*., 2003). The sPIF modulates integrins (crucial pro-implantation biomarkers in non-pregnant endometrial cells) expression, upregulates the expression and discharge of the pro-inflammatory ligands in HESCs. It finally asserts implantation by raising the secretions of amphiregulin, epiregulin and FGFs while diminishing proliferation promoter - betacellulin expression (Barnea *et al*., 2012a).

### Trophoblast invasion

Placentation as well as implantation is a key determinant of pregnancy success [30]. The ideal trophoblast invasion of the maternal spiral arteries can supply fetal oxygen and nutrient needs as poor invasion or excessive invasion, leading to obstetrics complications for the mother. PIF can produce a pro-tolerance milieu by enhancing both intracellular expression and surface expression levels of some HLA class-I such as HLA-G, HLA-E, and HLA-C in dose and time-dependent paths in cytotrophoblastic JEG-3 cells (Hakam *et al*., 2017). Therefore, trophoblast invasion can be used to balance the needs of the fetus that should be provided by the mother and protect the mother from such invasion (McFadyen, 1989; Anin *et al.,* 2004). This process that is regulated by matrix metalloproteinase (MMP) activity, alpha v, and alpha 1 integrin expressions (Moindjie *et al*., 2014) can facilitate trophoblast invasion either in vitro or in-vivo by PIF (Duzyj *et al*., 2010; Stamatkin *et al*., 2011a;b). Pro invasive or the positive regulatory effects of PIF in extravillous trophoblasts were associated with 1) the increasing of MMP9 activity, and 2) lower expression levels of tissue metalloproteinase-1 (TIMP1) inhibitor. The invasive function of PIF is found to be performed through the mitogen-activated protein kinase (MAPK), phosphoinositide-3-kinase (PI3K), Janus-kinase signal transducer and transcription (JAK-STAT) signaling pathways activator (Damsky *et al*., 1994; Staun-Ram *et al*., 2004; Knöfler, 2010). Taken together, PIF can be engaged in pathological pregnancies defined as incommensurate or extreme trophoblast invasion.

### Regulation of systemic immunity

PIF has dual effects in human peripheral blood mononuclear cells (PBMC), minimally affecting innate immunity (Barnea *et al*., 2012b). In other words, PIF/sPIF can attach to activated PBMCs, resulting in immune tolerance without suppression (Roussev *et al*., 2013), it also inhibits mixed lymphocyte reaction (MLR) extension in those cells, leading to a T helper 2 (Th2) cytokine bias while maintaining the T helper 1 (Th1) response, causing a remarkable decrease in macrophage penetrations. PIF also reduced the pro-inflammatory expression levels of adhesion molecules, cytokines, and chemokines in the plaque, also reducing circulating Interferon gamma (IFN-γ) (Chen *et al*., 2016). PIF directly regulates natural killer (NK) cell activity (Barnea *et al*., 2012b; Roussev *et al*., 2013; Barnea *et al*., 2016a). Low-dose PIF is efficient in NK cells toxicity reduction by down-regulating the expression levels of CD 69 (Roussev *et al*., 2013). The advanced regulatory effects of PIF on the PKC/PKA phosphorylation pathways was suppressed in the presence of Toll-like receptor 4 (TLR4) siRNA (Hoebe *et al*., 2003).

PIF operates on macrophages downstream of the lipopolysaccharides cluster definition 14 (LPS-CD14), TLR4, myeloid differentiation protein 2 (MD2) complex communicating with myosin-9, thymosin-α1 and 14-3-3 eta protein objects (Barnea *et al*., 2016a). Reports on LPS-activated macrophages indicated that PIF has the competence to reduce inducible nitric oxide synthase (iNOS2); in addition, the nitric oxide secretion revealing the protection against graft versus host disease development (Azar *et al*., 2013; Chen *et al*., 2016).

### PIF and pregnancy disorders

RPL is associated with several factors (anatomic, genetic, and hematologic abnormalities). Immune defects represent a major causing factor. Maternal circulating PIF, as well as the administration of sPIF, regulates systemic immunity, protects the embryo development and decreases circulating NK cells cytotoxicity in women with RPL (Christiansen, 2013). Accordingly, PIF deficiency negatively impacts on pregnancy success resulting in RPL; however, sPIF can modulate pregnancy outcome leading to a significantly reduced incidence of recurrent implantation failure and RPL (Kumar & Mahajan, 2013).

sPIF influences ectopic endometrial tissues of women with endometriosis by increasing the FoxP3 mRNA levels in ectopic endothelial cells, resulting in a significant decrease in Tregs of the patient's peripheral blood when compared to controls. On the contrary, the rate of Treg increased in the peritoneal fluid of those women suffering endometriosis (Olkowska-Truchanowicz *et al*., 2013). Therefore, the evidence reported by some researchers suggests that PIF expression, as a differential immune modulatory system, might mediate an immune privilege for endometriotic lesions (Olkowska-Truchanowicz *et al*., 2013; Sbracia *et al*., 2017).

Preeclampsia is a unique pregnancy disorder, with its pathophysiology beginning early in pregnancy, while its clinical manifestations usually occur in the middle to the late gestational age, and it should be effectively implemented at the beginning of pregnancy (Redman & Sargent, 2005). It is established that the PIF secreted early by viable embryos and then its interaction with its host-mother provided one of the potential mechanisms against preeclampsia (Barnea *et al*., 2016b).

Furthermore, PIF can prevent developmental disorders by modulating the uterine environment in the first trimester of pregnancy and also it can have a role in reducing the frequency of developing post-natal disorders (Duzyj *et al*., 2014).

### PIF and autoimmune disease (AD)

Rheumatoid arthritis (RA) and juvenile diabetes mellitus (JDM) are AD diseases positively affected in pregnancy with an improvement of patients symptoms in the first trimester until term (Tandon *et al*., 2006). In contrast, some AD disorders defined as Lupus, Crohn’s disease and ulcerative colitis are not affected by pregnancy unless they are diagnosed as severe diseases(Tincani *et al*., 2006; Cornish *et al*., 2007).

Some evidence points to the potential role of several factors; changing in some reproductive hormones levels, such as estrogen, and hCG increases the number of regulatory T cells (Treg), known to be involved in fighting AD, though these conflicting results suggest that these are not major participants in AD remission either (Langer-Gould *et al*., 2002; Sargent *et al*., 2006).

PIF has unique immune-modulatory properties, beyond pregnancy, for the prevention of ADs such as MS, and JDM, as well as preventing the development of graft-versus-host disease (GVHD) following semiallogeneic transplant in preclinical models (Barnea, 2007).

We know that PIF therapy could improve hematopoietic recovery and attenuate the production of systemic inflammatory cytokines after sub-lethal radiation exposure (Shainer *et al*., 2016). The sPIF analog was found to be a good representative with natural PIF action, mediated peripheral immune cells to achieve tolerance without immune suppression as a beneficial agent in autoimmune models in the nonpregnant models (Barnea, 2007; Than *et al*., 2007; Weiss *et al*., 2011).

The short-term administration of sPIF can prevent the progression of JDM in the diabetic mouse model through maintaining pancreatic islet function (Weiss *et al*., 2011). In the case of atherosclerosis, PIF is a strong immunomodulatory drug candidate for immune therapy as well as its prevention without affecting circulating lipids (Chen *et al*., 2016).

In GVHD, PIF diminished skin ulceration and colon ulceration as well as liver inflammation while preserving positive graft vs. leukemia effects (Azar *et al*., 2013).

## CONCLUSION

All clinical studies show that PIF has both local and systemic effects; it can create an integrated development environment for the treatment of various inflammation conditions, along with tackling developed disease.
